# Supplementary material for: The deglacial forest conundrum
Source: Nat Commun. 2022 Oct 13;13:6035. doi: 10.1038/s41467-022-33646-6 (PMC9562359; doi:10.1038/s41467-022-33646-6)
Supplement: Supplementary file 1 — Supplementary Information [file 41467_2022_33646_MOESM1_ESM.pdf]

Supplementary information for the manuscript "The deglacial forest conundrum"

Authors: Anne Dallmeyer[1*], Thomas Kleinen[1], Martin Claussen[1,2], Nils Weitzel[3,4], Xianyong Cao[5], Ulrike Herzschuh[6,7,8]

Affiliations:

[1]Max Planck Institute for Meteorology, Bundesstrasse 53, 20146 Hamburg, Germany

[2]Centrum für Erdsystemforschung und Nachhaltigkeit (CEN), Universität Hamburg, Bundesstrasse 55, 20146 Hamburg, Germany

[3]Institute of Environmental Physics, Heidelberg University, Im Neuenheimer Feld 229, 69120 Heidelberg, Germany

[4]Department of Geosciences, University of Tübingen, Schnarrenbergstr. 94-96, 72076 Tübingen, Germany

[5]Alpine Paleoecology and Human Adaptation Group (ALPHA), State Key Laboratory of Tibetan Plateau Earth System, and Resources and Environment (TPESRE), Institute of Tibetan Plateau Research, Chinese Academy of Sciences, Beijing, China.

[6]Alfred Wegner Institute, Helmholtz Centre for Polar and Marine Research, Potsdam, Germany.

[7]Institute of Environmental Sciences and Geography, University of Potsdam, Germany

[8]Institute of Biochemistry and Biology, University of Potsdam, Germany

***Correspondence to: anne.dallmeyer@mpimet.mpg.de**

Supplementary Figure 1

Comparison of simulated and reconstructed regional climate

The climate change simulated by MPI-ESM1.2 (black) is compared to the simulation TraCE-21ka[1] (grey) and to pollen-independent temperature reconstructions, reconstructions of temperature controlled d18O records **(a-e)**, and precipitation controlled d18O reconstructions **(f-j)** (red). For (a-e) differences to 0 ka, for (f-h) differences to 6ka, for (i,j) differences to 12ka are shown. Additional information on the records including their reference is given in Supplementary Table 1. The Last Glacial Maximum period (22 ka - 19 ka, light blue shading), Bølling/Allerød (14.6 ka – 12.9 ka, light red shading) and the Younger Dryas period (12.9 ka – 11.6 ka, light blue shading) are marked.

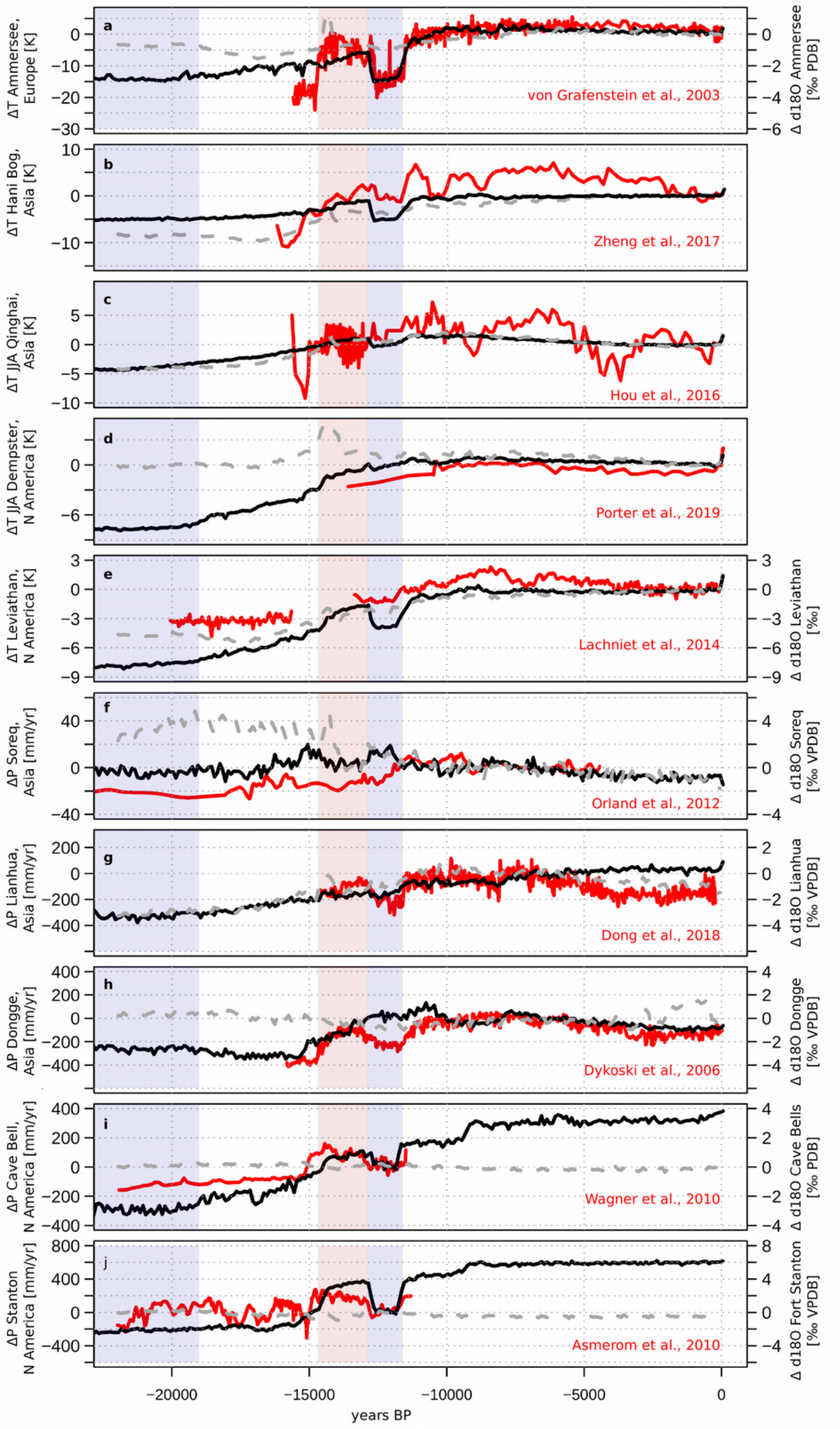

Supplementary Table 1: Information on the proxy records used for regional climate evaluation

Region	Location	Lat	Lon	Proxy	reference
Central Europe	Ammersee	47.1°N	11.02°E	Ostracod	Von Grafenstein et al.[2]
Northeast China	Hani Bog	42.22°N	126.52°E	glycerol dialkyl glycerol tetraether	Zheng et al.[3]
Tibetan Plateau	Qinghai Lake	36.81°N	100.14°E	alkenone	Hou et al.[4]
Eastern Beringia	Dempster Highway Peatland	65.21°N	138.32°W	d18O in ice	Porter et al.[5]
Southwestern United States	Leviathan	37.89°N	115.58°W	Speleothem d18O	Lachniet et al.[6]
Middle East	Soreq Cave	31.45°N	35.03 °E	Speleothem d18O	Orland et al.[7]
East Asian monsoon region	Lianhua Cave	38.4°N	113.8°E	Speleothem d18O	Dong et al.[8]
Southern China	Dongge Cave	25.28°N	108.08°E	Speleothem d18O	Dykoski et al.[9]
Southwestern United States	Cave of the bells	31.75°N	110.75°W	Speleothem d18O	Wagner et al.[10]
New Mexico	Fort Stanton	33.3°N	105.3°W	Speleothem d18O	Asmerom, et al.[11]

Supplementary Table 2: Gaussian kernel correlation test for the model-data agreement

The similarity between the MPI-ESM1.2 simulation, TraCE-21ka, and the proxy records is quantified by testing the similarity of the temporal patterns of the time series with a Gaussian kernel correlation (GKC). Correlations are calculated for **a)** the raw time-series and for oribtal- and **b)** millennial-scale temporal patterns which are separated with Gaussian smoothers[12] For the millennial scale, also the p-values are listed (in brackets). Due to the strong deglacial climate trend, the raw and orbital time-series have not enough degrees of freedom to calculate p-values. References for the records are given in Fig.1 or Supplementary Table 1.

**a)**		RAW			orbital scale	
	MPI-ESM & TraCE-21ka	MPI-ESM & Records	TraCE-21ka & Records	MPI-ESM & TraCE-21ka	MPI-ESM & Records	TraCE-21ka & Records
T NH (Shakun)	0.96	0.97	0.96	0.98	0.99	0.98
T NH (Osman)	0.96	0.99	0.98	0.98	0.98	0.99
P NH (Cariaco)	0.79	0.53	0.87	0.91	0.67	0.96
T Greenland	0.85	0.81	0.76	0.95	0.87	0.85
P Greenland	0.87	0.96	0.93	0.96	0.99	0.98
T Ammersee	0.76	0.94	0.66	0.92	0.96	0.86
T Hani	0.91	0.62	0.73	0.98	0.76	0.78
T Qinghai	0.97	0.4	0.5	0.99	0.79	0.77
T Dempster	0.44	0.59	0.29	0.57	0.18	-0.18
T Leviathan	0.96	0.96	0.93	0.98	1	0.97
P Soreq	0.21	-0.15	-0.58	0.28	-0.09	-0.78
P Lianhua	0.83	-0.12	0.66	0.87	-0.37	0.88
P Dongge	-0.44	0.45	0.02	-0.61	0.67	-0.22
P Cave Bell	-0.74	0.72	-0.4	-0.91	0.87	-0.67
P Stanton	-0.67	0.71	-0.49	-0.85	0.82	-0.84

**b)**		Millenial scale	
	MPI-ESM & TraCE-21ka	MPI-ESM & Records	TraCE-21ka & Records
T NH (Shakun)	0.52 (0.1)	0.79 (0.01)	0.69 (0.0)
T NH (Osman)	0.52 (0.1)	0.69 (0.0)	0.78 (0.0)
P NH (Cariaco)	0.52 (0.1)	0.5 (0.13)	0.77 (0.02)
T Greenland	0.43 (0.11)	0.48 (0.0)	0.56 (0.0)
P Greenland	0.23 (0.23)	0.68 (0.0)	0.6 (0.01)
T Ammersee	0.28 (0.19)	0.98 (0.0)	0.69 (0.0)
T Hani	0.4 (0.17)	0.33 (0.14)	0.58 (0.02)
T Qinghai	0.71 (0.01)	-0.01 (0.45)	0.26 (0.0)
T Dempster	0.29 (0.26)	0.29 (0.01)	0.37 (0.0)
T Leviathan	0.35 (0.18)	0.37 (0.19)	0.09 (0.41)
P Soreq	0.41 (0.07)	-0.3 (0.0)	0.03 (0.36)
P Lianhua	0.55 (0.01)	0.48 (0.0)	0.37 (0.0)
P Dongge	-0.1 (0.34)	0.22 (0.0)	-0.04 (0.26)
P Cave Bell	-0.3 (0.18)	0.83 (0.0)	-0.68 (0.0)
P Stanton	-0.46 (0.07)	0.62 (0.01)	-0.31 (0.13)

Supplementary Figure 2.

Record density for the last 22000 years

The temporal and spatial coverage of the records is displayed in form of **a)** the number of time steps available for each site (prepared with GMT 5.4.3[13]) and **b)** the number of available sites at each time-step, for the entire region (Northern Hemisphere > 30°N, total) and all individual continents. The entire analysis period covers 35 potential time steps. During the Holocene, temporal resolution is 500 years. During the deglaciation, temporal resolution is 1000 years[14].

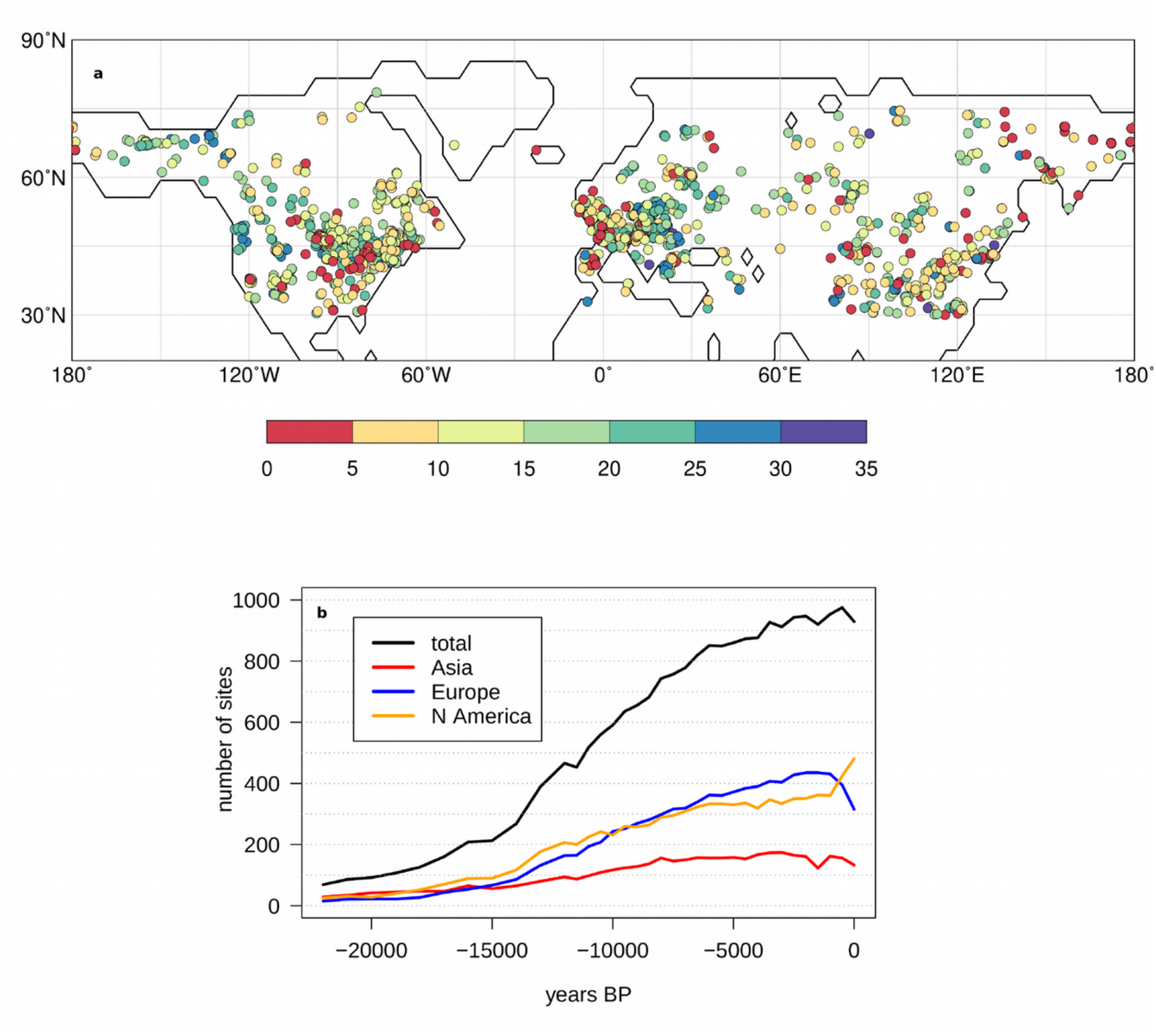

Supplementary Figure 3.

Climate difference between simulated 0ka time-slice and observations

Difference between the simulated 0ka climate and CRU TS3.10 data[15] (climatological mean of period 1960-1990), i.e. difference in **a)** mean temperature of the warmest month [K], **b)** mean temperature of the coldest month [K], and **c)** annual mean mean precipitation [mm/d]. The maps have been prepared with the software GMT, version 5.4.3[13].

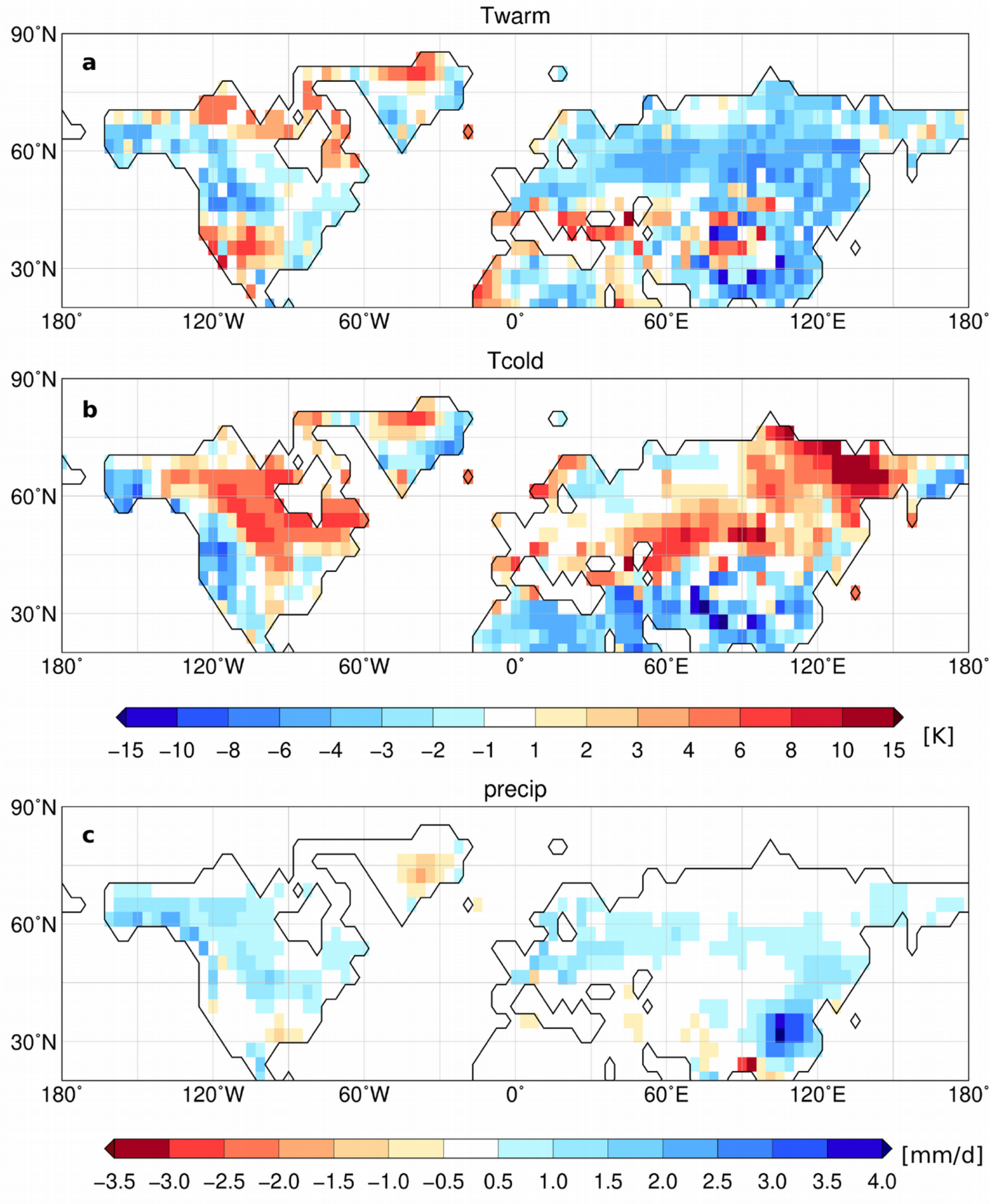

Supplementary Figure 4.

Comparison of pollen-based and non-pollen based moisture reconstructions for Asia

a) Pollen-based and **b)** non-pollen-based semi-quantitative moisture reconstructions for East Asia[16] are compared. Here, past moisture levels are categorized into "much wetter" (dark blue) or "wetter" (light blue) than at 0ka, "similar as" (grey) or "drier than" (yellow) at 0ka. Figure shows the percentage of all sites revealing the different moisture categories, respectively.

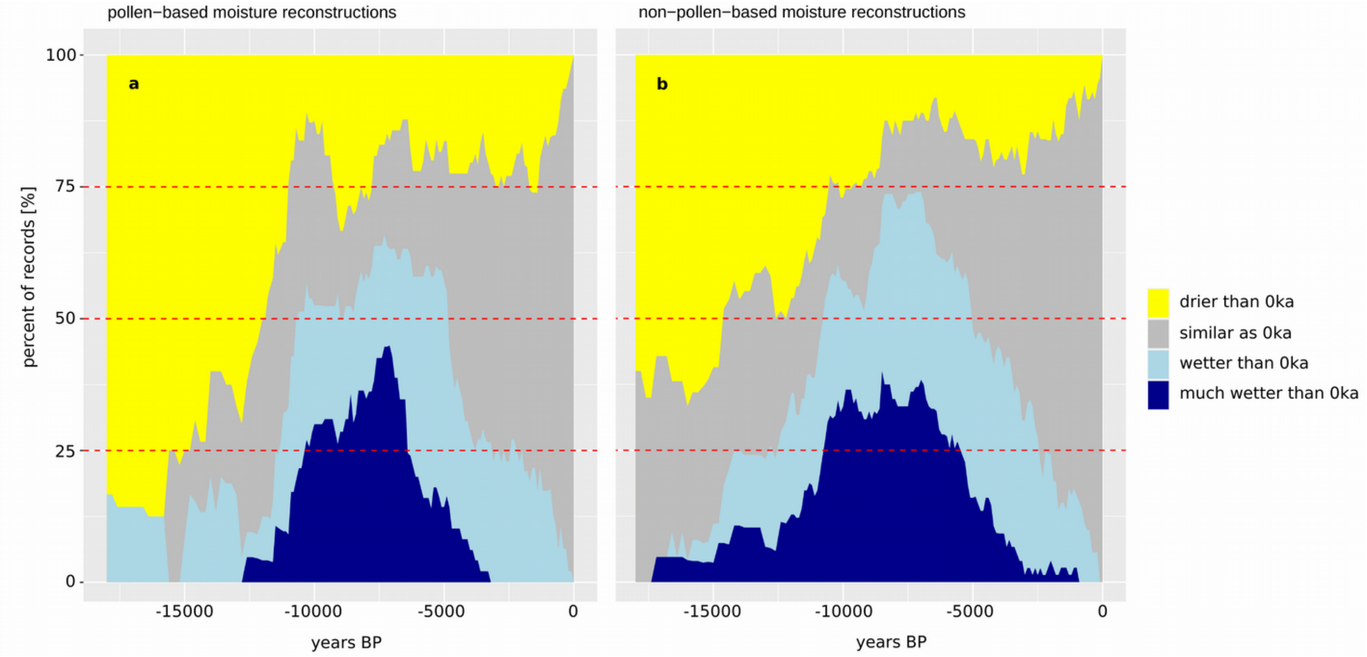

Supplementary Figure 5

The agreement between the simulated global biome distribution and BIOME6000 reconstructions with respect to other (time-slice) simulations for 21ka, 6ka and 0ka

Snapshots of the simulated MPI-ESM global biome distribution are compared to biome distributions calculated from other state-of-the art Earth System Model simulations, among them the PMIP3 simulations[17] with dynamic vegetation, taken from Dallmeyer et al.[18]. Both are evaluated against BIOME 6000 reconstructions[19] for 21ka, 6ka and a potential natural biome assessment for 0ka[20]. The agreement is displayed in terms of the fractional skill score (fss) for 0ka, and the Best neighbour score (BNS) for 6ka and 21ka for each individual mega-biome and in total. For details on the metrics and simulations we refer to Dallmeyer et al.[18].

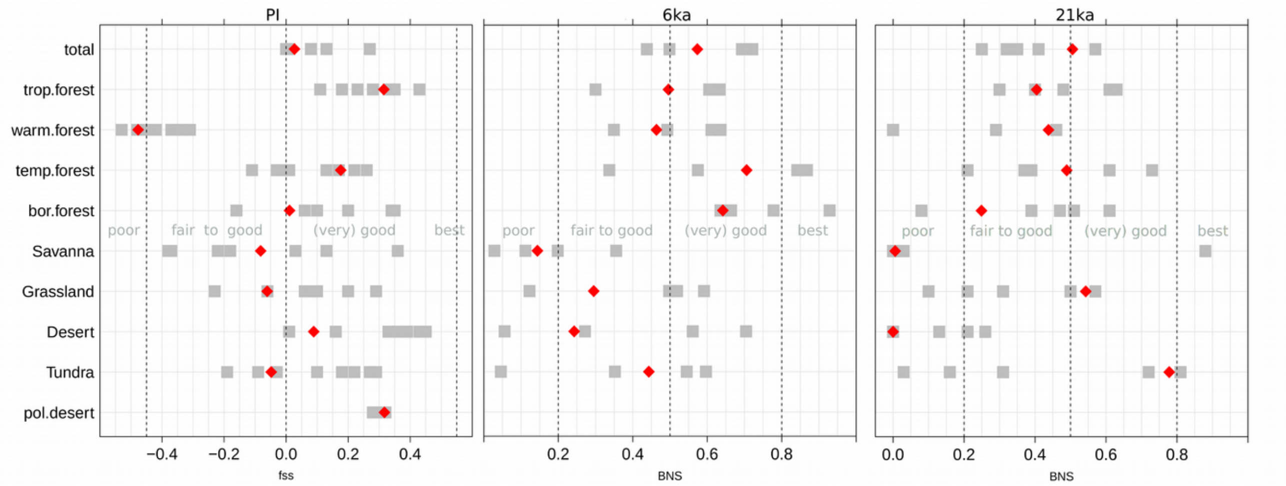

Supplementary References:

1. He, F. Simulating transient clmate evolution of the last deglaciation with CCSM3 (2011).
2. Von Grafenstein, U., Erlenkeuser, H., Brauer, A., Jouzel, J. & Johnsen, S. J. A mid-European decadal isotope-climate record from 15,500 to 5000 years B.P. *Science (80-.).* **284**, 1654–1657 (1999).
3. Zheng, Y. *et al.* Atmospheric connections with the North Atlantic enhanced the deglacial warming in northeast China. *Geology* **45**, 1031–1034 (2017).
4. Hou, J. *et al.* Large Holocene summer temperature oscillations and impact on the peopling of the northeastern Tibetan Plateau. *Geophys. Res. Lett.* **43**, 1323–1330 (2016).
5. Porter, T. J. *et al.* Recent summer warming in northwestern Canada exceeds the Holocene thermal maximum. *Nat. Commun.* **10**, 1–10 (2019).
6. Lachniet, M. S., Denniston, R. F., Asmerom, Y. & Polyak, V. J. Orbital control of western North America atmospheric circulation and climate over two glacial cycles. *Nat. Commun.* **5**, 1–8 (2014).
7. Orland, I. J. *et al.* Seasonal resolution of Eastern Mediterranean climate change since 34 ka from a Soreq Cave speleothem. *Geochim. Cosmochim. Acta* **89**, 240–255 (2012).
8. Dong, J. *et al.* Rapid retreat of the East Asian summer monsoon in the middle Holocene and a millennial weak monsoon interval at 9 ka in northern China. *J. Asian Earth Sci.* **151**, 31–39 (2018).
9. Dykoski, C. A. *et al.* A high-resolution, absolute-dated Holocene and deglacial Asian monsoon record from Dongge Cave, China. *Earth Planet. Sci. Lett.* **233**, 71–86 (2005).
10. Wagner, J. D. M. *et al.* Moisture variability in the southwestern United States linked to abrupt glacial climate change. *Nat. Geosci.* **3**, 110–113 (2010).
11. Asmerom, Y., Polyak, V. J. & Burns, S. J. Variable winter moisture in the southwestern United States linked to rapid glacial climate shifts. *Nat. Geosci.* **3**, 114–117 (2010).
12. Rehfeld, K., Marwan, N., Heitzig, J. & Kurths, J. Comparison of correlation analysis techniques for irregularly sampled time series. *Nonlinear Process. Geophys.* **18**, 389–404 (2011).
13. Wessel, P. et al. Generic Mapping Tools: Improved Version Released, *EOS Trans. AGU*, 94(45), p. 409–410, (2013).
14. Cao, X., Tian, F., Dallmeyer, A. & Herzschuh, U. Northern Hemisphere biome changes (>30°N) since 40 cal ka BP and their driving factors inferred from model-data comparisons. *Quat. Sci. Rev.* **220**, 291–309 (2019).
15. Harris, I., Jones, P. D., Osborn, T. J. & Lister, D. H. Updated high-resolution grids of monthly climatic observations - the CRU TS3.10 Dataset. *Int. J. Climatol.* **34**, 623–642 (2014).

16. Wang, Y. *et al.* Coherent tropical-subtropical Holocene see-saw moisture patterns in the Eastern Hemisphere monsoon systems. *Quat. Sci. Rev.* **169**, 231–242 (2017).

17. Braconnot, P. *et al.* Evaluation of climate models using palaeoclimatic data. *Nature Climate Change* vol. 2 417–424 (2012).

18. Dallmeyer, A., Claussen, M. & Brovkin, V. Harmonising plant functional type distributions for evaluating Earth system models. *Clim. Past* **15**, 335–366 (2019).

19. Harrison, S. BIOME 6000 DB classified plotfile version 1. (2017) doi:10.17864/1947.99.

20. Ramankutty, N. & Foley, J. A. Estimating historical changes in global land cover: Croplands from 1700 to 1992. *Global Biogeochem. Cycles* **13**, 997–1027 (1999).
